# The evaluation of a non-invasive respiratory monitor in ards patients in supine and prone position

**DOI:** 10.1007/s10877-024-01147-0

**Published:** 2024-03-26

**Authors:** Tommaso Pozzi, Silvia Coppola, Elena Chiodaroli, Federico Cucinotta, Francesca Becci, Davide Chiumello

**Affiliations:** 1 Department of Health Sciences, University of Milan, Milan, Italy; 2 Department of Anesthesia and Intensive Care, ASST Santi Paolo e Carlo, San Paolo University Hospital, Milan, Italy; 3 Coordinated Research Center on Respiratory Failure, University of Milan, Milan, Italy

**Keywords:** ARDS, Respiratory monitoring, Non-invasive monitoring, Electrical impedence, Acute respiratory failure

## Abstract

Purpose: The Prone positioning in addition to non invasive respiratory support is commonly used in patients with acute respiratory failure. The aim of this study was to assess the accuracy of an impedance-based non-invasive respiratory volume monitor (RVM) in supine and in prone position. Methods: In sedated, paralyzed and mechanically ventilated patients in volume-controlled mode with acute respiratory distress syndrome scheduled for prone positioning it was measured and compared non-invasively tidal volume and respiratory rate provided by the RVM in supine and, subsequently, in prone position, by maintaining unchanged the ventilatory setting. Results: Forty patients were enrolled. No significant difference was found between measurements in supine and in prone position either for tidal volume (*p* = 0.795; *p* = 0.302) nor for respiratory rate (*p* = 0.181; *p* = 0.604). Comparing supine vs. prone position, the bias and limits of agreements for respiratory rate were 0.12 bpm (-1.4 to 1.6) and 20 mL (-80 to 120) for tidal volume. Conclusions: The RVM is accurate in assessing tidal volume and respiratory rate in prone compared to supine position. Therefore, the RVM could be applied in non-intubated patients with acute respiratory failure receiving prone positioning to monitor respiratory function.

## Introduction

vThe monitoring of respiratory function in multiple clinical settings - such as in the intensive care unit, in the emergency department and in the perioperative period - is commonly based on the clinical examination of the patient, on the assessment of respiratory rate and on the measurement of peripheral saturation by pulse oximetry [1]. Although respiratory rate is also included in the Early Warning Score, alerting the physician when it is higher or lower compared to a predefined range [2], it cannot be used as a surrogate for minute ventilation for detecting episodes of hypoventilation characterized by reduced tidal volume and normal respiratory rate [2]. Pulse oximetry is the most common technique for oxygenation monitoring to detect hypoxic respiratory failure. However, oximetry is a late indicator of respiratory depression as it does not directly measure alterations in minute ventilation; moreover, the use of supplemental oxygen could mask an underlying respiratory depression [3]. In addition, continuous monitoring by pulse oximetry did not show to reduce admission rate in intensive care unit and the overall mortality [3]. A relatively new respiratory volume monitor (RVM) based on the changes in the thoracic impedance measured through electrode PadSets placed on the patients’ chest surface has been released [4, 5]. This totally non-invasive RVM is able to continuously display both respiratory rate and tidal volume in a real-time fashion. Previous data showed that this RVM can detect respiratory failure before hypercapnia or hypoxemia occur [6]. Voscopoulos et al. showed that the measurement of respiratory rate and tidal volume in spontaneous breathing subjects over a period of 24 h by RVM technique presented an average error lower than 10% compared with spirometer-derived values [4]. In a subsequent study, the same group of authors evaluated the relationship between the measurement of tidal volume and respiratory rate between the RVM monitoring and a mechanical ventilator in intubated patients without cardiopulmonary diseases [6]; the RVM showed a good accuracy compared to the mechanical ventilator, both during controlled mechanical ventilation and the weaning phase. During non-invasive respiratory support, such as continuous positive airway pressure (CPAP) or high-flow nasal cannula, it has been proposed to assess inspiratory effort by a composite score made up by respiratory rate and the visual assessment of chest excursion and inspiratory muscle activity [7]; however, these indicators can only be obtained periodically and are often inaccurate [8]. Therefore, the measurement of tidal volume, in addition to respiratory rate, could provide a more comprehensive assessment of respiratory status and improve the detection rate of possible deterioration of respiratory function. Furthermore, prone positioning is more and more often applied also during non-invasive respiratory support, since COVID-19 pandemia, to improve outcome. Thus, a reliable assessment of respiratory rate and tidal volume is necessary not only in supine, but also in prone position. Currently there is a lack of data regarding the accuracy of impedance-based RVMs in prone compared to supine position. The aim of this study is to evaluate the accuracy of the measurement of respiratory rate and tidal volume in supine and prone position by the impedance-based RVM in mechanically ventilated ARDS patients.

## Materials and methods

### Study design

Sedated, paralyzed and mechanically ventilated ARDS patients admitted to the intensive care unit (ICU) of the ASST Santi Paolo Carlo Hospital, Milan, Italy and scheduled for prone positioning due to the severity of hypoxemia (PaO_2_/FiO_2_ < 150) were consecutively enrolled. Exclusion criteria were: history of chronic obstructive pulmonary disease (COPD) and previous lung surgery. The study was approved by the Institutional Review Board of our hospital (Comitato Etico Interaziendale Milano Area A, 2023/ST/057) and informed consent was obtained according to the Italian regulations.

### Data collection

A non-invasive impedance-based respiratory volume monitor (RVM; ExSpiron, Senzime Inc., Watertown, MA, USA) was applied to continuously measure tidal volume and respiratory rate. With patients laying in supine position, the electrode PadSet was positioned on the anterior thorax surface at the sternum notch, xyphoid and at the right mid-axillary line at the level of the xyphoid. Holding ventilatory setting unchanged, three consecutive measurements of tidal volume and respiratory rate were collected both from the RVM and from the mechanical ventilator in three 5-minutes steps. The electrode SetPad was left in place when the patients were placed in prone position; after a minimum of 2 h since the maneuver, the same set of measurement were taken. Ventilatory settings remained unchanged from supine to prone position.

### Statistical analysis

We calculated that a sample size of 40 patients would ensure the study a power of 0.80 with a confidence level of 0.05 to detect any difference in tidal volume or respiratory rate from the supine to the prone position. Continuous data are expressed as median [IQR], while categorical data are expressed as percentage (number). To assess the repeatability of the measurement, both in supine and in prone position, One-Way Repeated Measures ANalysis Of VAriance (ANOVA) or Friedman Test were used, as appropriate. A linear model was used to assess the association between variables measured in supine and in prone position. A Bland-Altman analysis was performed to assess the accuracy of the measurements taken in prone with respect to supine position; the latter was considered as the reference method for comparison. A *p* value less than 0.05 was considered as statistically significant. The analyses were performed using R (R Foundation for Statistical Computing, Vienna, Austria) and RStudio (RStudio, PBC, Boston, MA).

## Results

Forty patients were enrolled. The main clinical characteristics of the whole population are shown in Table 1. All patients were ventilated in volume-controlled ventilation. Tidal volume and respiratory rate and were 460 [440–510] mL and 16 [15–17] bpm, both in supine and in prone position.

**Table 1:** Baseline characteristics of the study population in supine position, before prone positioning

	*n* = 40
Age, *years*	68 [60–72]
Male sex, % *(n)*	65 (26)
Weight, *kg*	82 [70–90]
Height, *cm*	175 [168–176]
Body mass index, *kg/m*^*2*^	27.5 [25.8–30.4]
SAPS II	42 [35–45]
SOFA score	3 [2–7]
Charlson comorbidity score	5 [3–5]
*Sedative setting*	
Propofol, *mg/kg/h*	3.4 [2.3–4.2]
Midazolam, *mg/kg/h*	0.07 [0.04–0.08]
Fentanyl, *µg/kg/h*	2.1 [1.4–2.8]
Rocuronium, *mg/kg/h*	0.4 [0.3–0.6]
*Ventilatory setting*	
Tidal volume, *mL*	460 [440–510]
Respiratory rate, *bpm*	16 [15–17]
PEEP, *cmH*_*2*_*O*	10 [8–10]
Inspired oxygen fraction	0.70 [0.60–0.90]
*Gas exchange*	
PaO_2_, *mmHg*	76 [62–84]
PaCO_2_, *mmHg*	48 [42–58]
PaO_2_/FiO_2_ ratio	97 [79–145]

**Fig. 1:**
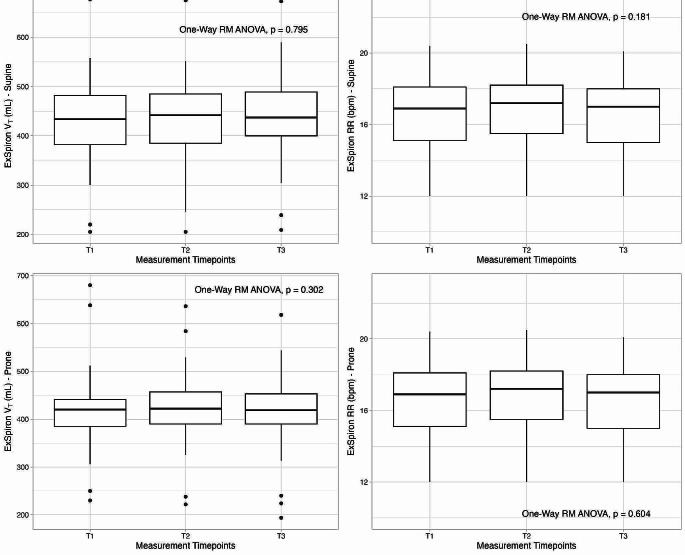
Repeatability analysis for tidal volume **(A, C)** and respiratory rate **(B, D)** in supine and prone position, respectively, collected in three measurement timepoints (T1, T2 and T3) at identical respiratory setting by the impedance-based respiratory volume monitor

In Fig. 1 the repeatability analysis for tidal volume (A, C) and respiratory rate (B, D) in supine and prone position is reported. No significant difference was found among measurement timepoints either for tidal volume (supine, *p* = 0.795; prone, *p* = 0.302) nor for respiratory rate (supine, *p* = 0.181; prone, *p* = 0.604) in both positions.

**Fig. 2:**
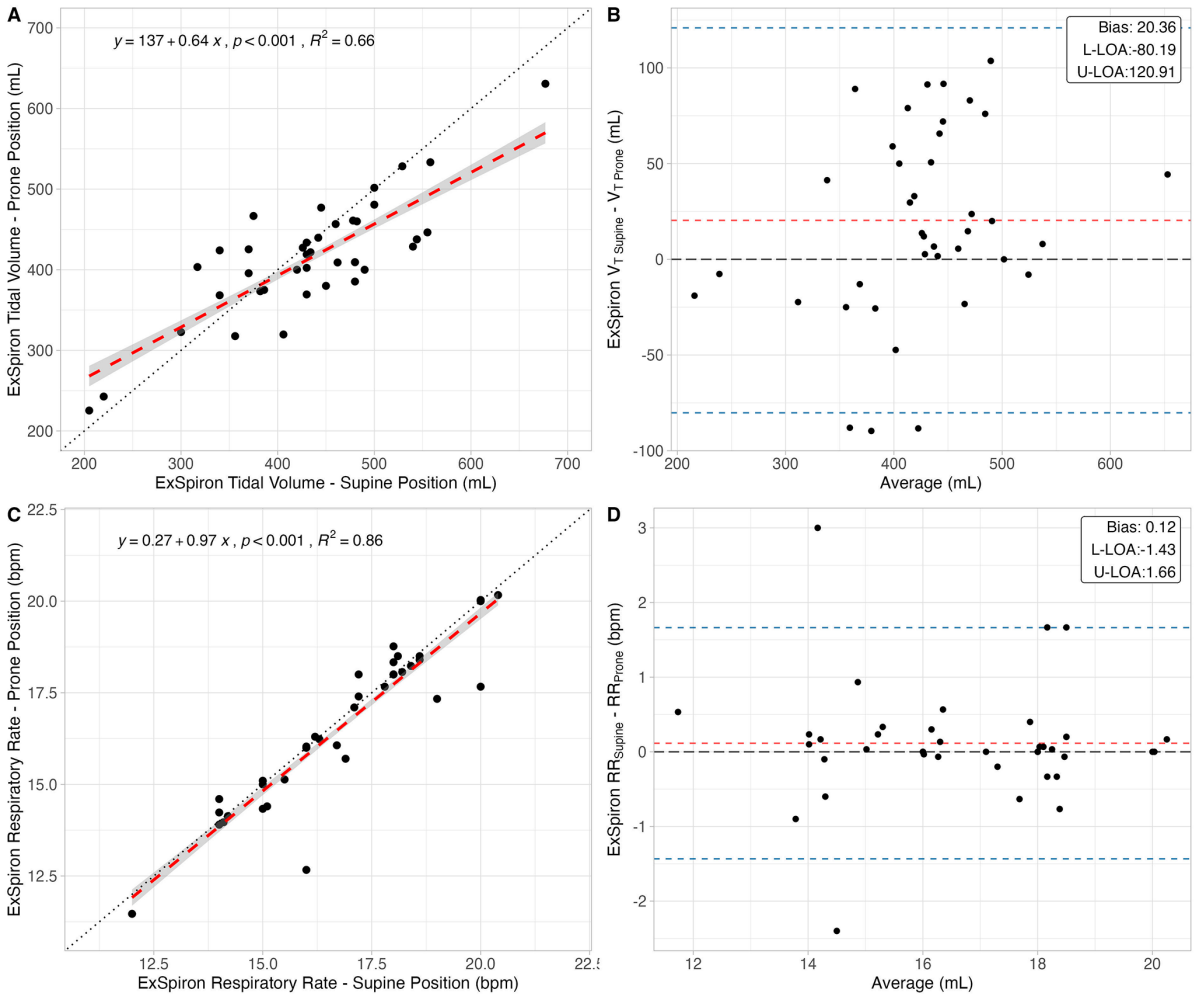
Accuracy analyses, consisting of linear regressions (left) and Bland-Altman analyses (right), of tidal volume (upper panels) and respiratory rate (lower panels) measured by the impedance-based respiratory volume monitor in prone vs. supine position. L-LOA: lower limit of agreement; U-LOA: upper limit of agreement

In Fig. 2 the linear regressions (A, C) and Bland–Altman analyses (B, D) for respiratory rate and tidal volume between prone and supine position (assumed as the reference method) are reported. Tidal volume and respiratory rate measured in prone position were well and almost perfectly associated with the values measured in supine position, respectively (R^2^ = 0.66 and R^2^ = 0.86). Considering tidal volume between supine and prone position, the bias was 20 mL with limits of agreements from − 80 to 120 mL. The bias for respiratory rate was 0.12 bpm, with limits of agreement from − 1.4 to 1.6 bpm.

In Table 2a summary of the agreement analysis is reported.

**Table 2:** Summary of the results of the accuracy analysis for tidal volume and respiratory rate in prone vs. supine position measured by the impedance-based respiratory volume monitor. L-LOA: lower limit of agreement; U-LOA: upper limit of agreement

	*p*	R^2^	Bias	L-LOA	U-LOA
Tidal volume, *mL*	*< 0.001*	0.66	20.4	-80.2	120.9
Respiratory rate, *bpm*	*< 0.001*	0.86	0.1	-1.4	1.7

## Discussion

In the present study, the impedance-based RVM showed a clinically-acceptable accuracy in assessing both tidal volume and respiratory rate in prone compared to supine position in mechanically ventilated ARDS patients. This finding supports the possibility to use this system to monitor minute ventilation during non-invasive respiratory support in prone position.

The standard non-invasive respiratory monitoring for critically ill patients admitted in intensive care unit and during perioperative period is commonly based on an intermittent assessment of respiratory rate and continuous peripheral saturation measurement through pulse oximetry. However, these indicators can miss early warning signals for respiratory failure in terms of inadequate tidal volume [9].

Although widely used, pulse oximetry can detect already-deteriorated states of respiratory function rather than alert the physician before respiratory derangement occurs. In fact, respiratory failure often begins with changes in minute ventilation rather than with desaturation, which is commonly assessed using pulse oxymeters [10–13]. Furthermore, it has been reported that only up to 40% of the physician is correctly aware of the fact that pulse oximetry does not reflect changes in minute ventilation [14]. Furthermore, a recent survey of closed anesthesia malpractice of claims evaluating respiratory depression reported that up to 80% of these events occurred within 24 h after surgery and 13% within 2 h after arriving in the general ward [15]. The majority of these claims are thought to be preventable with a better monitoring of the patients [15].

Adequate ventilation monitoring is essential in different clinical scenarios, such as during the application of non-invasive respiratory support (CPAP and HFNC), as well in post-operative patients, in order to detect the deterioration of respiratory function. Respiratory rate alone could be not related to minute ventilation; thus, it could not detect hypoventilation as well as hyperpnea [16]. In first few hours after surgery, the absence of correlation between respiratory rate and tidal volume has been reported: while respiratory rate significantly increased, tidal volume did not change [17]. Similarly, during non-invasive ventilatory support, the assessment of minute ventilation presented better accuracy in detecting respiratory impairment compared to respiratory rate alone [18]. Thus, a continuous non-invasive assessment of respiratory status in terms of tidal volume and respiratory rate in spontaneous breathing patients and during non-invasive respiratory support could improve the outcome during respiratory failure by an earlier detection of hyper-/hypo-capnia [19]. In addition, during CPAP and high flow nasal canula it is not possible to measure tidal volume, which has been previously showed to be associated to a risk for possible respiratory failure and worse outcome [20].

The RVM is an automated non-invasive continuous monitor of respiratory function based on changes of chest electrical impedance occurring during the respiratory cycle [4–6], which is able to measure tidal volume and respiratory rate [5]. Actually, the RVM is not able to directly measure tidal volume, but tidalic impedance changes; thus, it is most reliable when used to monitor relative changes over time.

Previous data showed that RVM has been successfully applied in different clinical scenarios to assess respiratory function in not intubated patients, reducing the risk of under-detection of respiratory depression [9, 10]. In 259 surgical patients, the RVM detected respiratory depression up to 10 min before the onset of desaturation [21].

Thus, the RVM could be applied in the perioperative setting for a continuous non-invasive monitoring of respiratory function, improving the patient safety by detecting an early respiratory deterioration and minimizing respiratory complications. Furthermore, being non-invasive ventilatory support more frequently used in prone position nowadays, the RVM could be also applied in this position for a continuous monitoring of respiratory function.

Voscopoulos et al. evaluated the accuracy of RVM compared to standard spirometer in different breathing conditions in a small group of healthy spontaneous breathing subjects [4], showing a relative error less than 10%. In intubated, mechanically ventilated patients under general anesthesia, the RVM measurements were compared to ventilator measurements [22], demonstrating the same accuracy. In intubated post cardiac surgery with a median sternotomy, tidal volume and respiratory rate obtained by the RVM were well correlated with those measured by the mechanical ventilator [23]. However, these data were analyzed only in supine position. At the present time, there are no data assessing the accuracy of RVM in prone position.

In the present study, a similar accuracy was shown when comparing supine and prone position measurement of tidal volume and respiratory rate. However, there was a slightly higher confidence interval for tidal volume compared to previous data [6]. It is worth noting that patients in the present study fulfilled ARDS criteria, which is characterized by lung edema, posterior atelectasis and increase in lung weight; these characteristics can significantly modify thoracic impedance when moving from supine to prone position [24, 25]. Thus, the RVM was demonstrated to be reliable and accurate also in the context of an inhomogeneous pulmonary disease.

In a previous study RVM was applied to monitor the global minute ventilation in supine spontaneous breathing patients affected with COVID-19 respiratory failure and its association with mortality [26]. Basing on the present results, the RVM could be used in clinical practice for the continuous monitoring of spontaneously breathing patients with and without respiratory failure both in supine and in prone position. This is especially important since, traditionally, only respiratory rate and oxygen saturation are routinely assessed in these patients, while tidal volume monitoring can represent an early indicator for patients at risk of respiratory failure.

## Conclusions

The RVM is accurate in assessing tidal volume and respiratory rate in prone as compared to supine position in ARDS patients. Therefore, the RVM could be applied in non-intubated patients with acute respiratory failure receiving prone positioning to monitor respiratory function.

## Data Availability

No datasets were generated or analysed during the current study.
